# SW-actors: accelerating the Smith–Waterman algorithm via actors

**DOI:** 10.1093/bioadv/vbaf173

**Published:** 2025-07-28

**Authors:** Reza Rafati Bonab, Ali Akbar Jamali, Kyle Klenk, Mohammad Mahdi Moayeri, Raymond J Spiteri

**Affiliations:** Department of Computer Science, University of Saskatchewan, Saskatoon, SK S7N 5C9, Canada; Department of Computer Science, University of Saskatchewan, Saskatoon, SK S7N 5C9, Canada; Department of Computer Science, University of Saskatchewan, Saskatoon, SK S7N 5C9, Canada; Department of Computer Science, University of Saskatchewan, Saskatoon, SK S7N 5C9, Canada; Department of Computer Science, University of Saskatchewan, Saskatoon, SK S7N 5C9, Canada

## Abstract

**Motivation:**

The Smith–Waterman (SW) algorithm is widely regarded as the gold standard for local sequence alignment. However, its time complexity in a serial implementation limits its practicality for large datasets. In this article, we introduce SW-actors, a parallel implementation of the SW algorithm that leverages the actor model of concurrent computation to optimize resource utilization by efficiently scheduling and managing independent alignment tasks across processors at both the interalignment and intraalignment levels.

**Results:**

SW-actors is compared with the state-of-the-art implementations Parasail, SeqAn, and SWIPE using four datasets of varying sequence lengths ranging from 85 to 74778 nucleotides. In terms of wall-clock time, SW-actors is 1.33×, 2.00×, 2.49×, and 1.94× faster than the next best implementation for the different datasets. SW-actors is up to 22× faster than serial on 40 cores. The speedup is consistent for larger datasets and hence offers significant advantages for medium- to large-scale alignments.

**Availability and implementation:**

The SW-actors source code and underlying data are available at https://git.cs.usask.ca/numerical_simulations_lab/actors/papers/sw-actors.

## Introduction

High-throughput structural genomics and genome sequencing have led to extensive datasets of DNA, RNA, and protein structures and sequences, offering researchers tremendous opportunities to make new discoveries. This surge in data, however, also presents a significant challenge to extract relevant insights within acceptable timeframes (Fang *et al.* 2023, Ghoussaini *et al.* 2023, Porubsky and Eichler 2024). Interdisciplinary collaborations between researchers in biology and computer science are emerging to address this challenge (Bentley 2002, Cechova 2020). These efforts have led to development of various bioinformatics algorithms that analyze biological sequences to decipher genomic codes, elucidate functionality, and predict structures solely from sequence information (Zhang *et al.* 2024).

Identification of similarities between biological sequences is a valuable operation in bioinformatics (Pearson 2013). Pairwise sequence alignment aims to identify regions of similarity between a *query* sequence and a subject or *database* sequence (Caucchiolo and Cicalese 2023) among two or more DNA, RNA, or protein sequences.

Sequence alignment is commonly categorized as global or local. Global sequence alignment aims to align as many characters as possible between the query sequence and the database sequence. In contrast, local sequence alignment identifies short stretches of similarity between two sequences (Barton *et al.* 2015). The Needleman–Wunsch (NW) (Needleman and Wunsch 1970) and Smith–Waterman (SW) (Smith and Waterman 1981) algorithms are mostly used methods for global and local sequence alignment, respectively. Almost all the applications of new sequencing technologies, however, are based on local sequence alignment, and the SW algorithm is generally regarded as the gold standard in many of these applications. In high-throughput sequencing, the SW algorithm is frequently used to align sequencing reads to reference sequences (Łukasz Ligowski *et al.* 2011). Identifying the optimal alignment using the SW algorithm is computationally expensive due to its time complexity: it performs an exhaustive search to find the optimal local alignment between two sequences (Beretta and Dondi 2024). This high computational cost has led to the development of various heuristic algorithms that are built upon the principles of the SW algorithm. For instance, tools like basic local alignment search tool (BLAST) and its variants utilize heuristic methods to significantly speed up the search process by focusing on key sequence matches and reducing exhaustive comparisons (Camacho *et al.* 2009, 2023).

Despite significant progress in enhancing the performance of sequence alignment techniques, there is a continued need for improvement, especially with respect to handling large-scale datasets and multiple alignments. As has been done in various other fields (Penas *et al.* 2015, Asgari *et al.* 2022, Maksum *et al.* 2022), parallel computing has been used to improve the performance of computationally intensive bioinformatics algorithms (Banimfreg 2023, López-Fernández *et al.* 2024, Schmidt and Hildebrandt 2024).

This study demonstrates that the SW algorithm can be accelerated at both the interalignment and intraalignment levels using actors. The actor model is a theoretical framework for concurrent computation that has gained considerable attention for its elegant approach to managing concurrent processes (Hewitt *et al.* 1973). In this model, individual computational entities (or “actors”) encapsulate both state and behavior, communicating solely through asynchronous message passing. Each actor operates independently, allowing for decentralized control and eliminating the need for shared memory and thus inherently avoiding the risk of deadlock and race conditions. The actor model also supports fault tolerance and scalability because actors can dynamically spawn new actors and distribute work across processors via work stealing. The actor model is applied in various domains, including cloud computing, real-time systems, and video games, where they offer a versatile solution for building highly concurrent and responsive systems (Bernstein and Bykov 2016, Bernstein *et al*. 2017, Srirama *et al*. 2021). More recently, the use of actors has demonstrated notable utility in scientific computing (Klenk *et al.* 2024, Klenk and Spiteri 2024). Similarly for sequence alignment, the actor model is a promising approach due to its ability to handle large-scale data processing tasks efficiently and concurrently. By representing each sequence alignment task as an independent actor, parallelism can be leveraged to accelerate computations in a scalable fashion. In this study, we compare the results of an actors-based implementation of the SW algorithm with other state-of-the-art Message-Passing Interface (MPI)- and OpenMP-based implementations to perform local sequence alignment using four datasets of varying sizes, both in terms of the number and length of sequences.

## Background

### SW algorithm

The SW algorithm is a local sequence alignment algorithm based on dynamic programming to compare two or more sequences. For a pairwise sequence alignment with two sequences *p* = $p_{1},p_{2},\ldots ,p_{m}$ and *q* = $q_{1},q_{2},\ldots ,q_{n}$ with lengths *m* and *n*, respectively, the computational complexity of the algorithm is O(mn). Three parameters, *match*, *mismatch*, and *gap*, are used to assign scores to determine an optimum sequence alignment of biological sequences. The algorithm can be divided into two phases: computation of the score matrix and backtracking.

#### Computation of the score matrix

The first phase of the SW algorithm computes a score matrix with elements $H_{i,j}$ for i=1,2,…,m, j=1,2,…,n, according to the recursion,

(1) $$H_{i,0}=0,$$

 

(2) $$H_{0,j}=0,$$

 

(3) $$H_{i,j}=\max\{\begin{array}{c} 0,\\ H_{i-1,j-1}+\text{match}(p_{i},q_{j}),\\ E_{i,j},\\ F_{i,j}, \end{array}$$

 

(4) $$E_{i,j}=\max\{\begin{array}{c} H_{i,j-1}-G_{\text{oe}},\\ E_{i,j-1}-G_{e}, \end{array}$$

**Figure 1.**
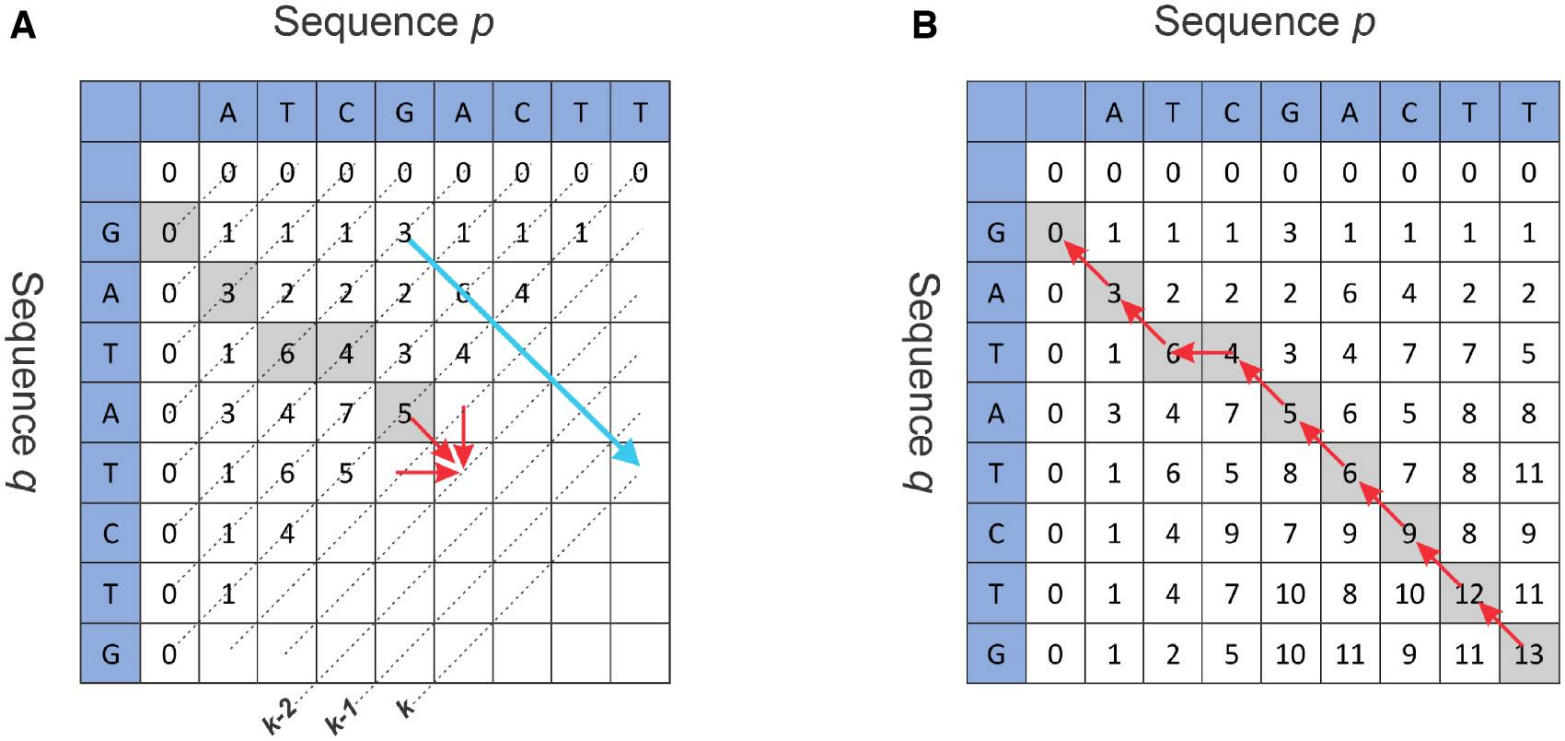
(A) The computation phase of the Smith–Waterman algorithm. The red arrows show the data dependence. The computation progresses diagonally, moving from the top left to the bottom right. (B) The backtracking phase of the Smith–Waterman algorithm. The computation starts from the cell with the highest score and ends when it reaches the element in the matrix below a preset threshold value.

 

(5) $$F_{i,j}=\max\{\begin{array}{c} H_{i-1,j}-G_{\text{oe}},\\ E_{i-1,j}-G_{e}, \end{array}$$

where $H_{i,j}$ represents the score for aligning the sequences *p* and *q* ending at positions *i* and *j*, respectively. $E_{i,j}$ and $F_{i,j}$ are the scores ending with a gap involving the first *i* and *j* residues of *p* and *q*, respectively. $G_{\text{oe}}$ is the sum of the opening gap (introducing an initial gap in one or both aligned sequences) and the extension gap (extending an initial opening gap) penalties, and $G_{e}$ is the extension gap penalty. The maximal alignment score in the matrix *H* represents the optimal local alignment score, denoted by *S*. It is noteworthy that $H_{i,j}$ depends only on the three neighboring cells, $H_{i,j-1}$, $H_{i-1,j}$, and $H_{i-1,j-1}$, as depicted in Fig. 1A. Consequently, the computation proceeds diagonally from the top left to the bottom right. Accordingly, all elements along antidiagonal *k* can be simultaneously calculated using antidiagonals k−2 and k−1, as depicted in Fig. 1A. For example, given two DNA sequences *p* = ATCGACTT and *q* = GATATCTG to be aligned, the first row and column represent nucleotides of sequence *p* and *q*, respectively. The second row and column are initialized to 0. Now taking match=3, mismatch=1, and gap=−2, we obtain the numerical values given in Fig. 1A.

#### Backtracking

The backtracking phase follows the computation of the score matrix, as depicted in Fig. 1B. Backtracking begins by initializing the starting point to the cell in matrix *H* that corresponds to *S*. Subsequently, the next backtracking position is the element from which the current result was calculated—either up, left, or upper-left. Backtracking continues until the element with the threshold value (typically taken to be 0) is reached. Ultimately, the backtracking path forms the optimal alignment.

### Actor model

#### Architecture

The actor model was first proposed in 1973 by Hewitt *et al.* (1973) and later extended by Agha (1985) in 1985 as a theoretical model of concurrent computation. In this model, actors are independent entities of computation that communicate exclusively through asynchronous messages (i.e., individual actors do not share state). Agha extended Hewitt’s original theory by formalizing the actions that an actor can perform upon receiving a message, namely, *send*, *create*, and *become*. When an actor receives a message, it can *send* messages to other actors, *create* new actors, or use the *become* operation to change its own behavior.

To facilitate the implementation of the actor model, current implementations, such as the C++ Actor Framework (CAF) (Charousset *et al.* 2014), often include additional features and data structures not present in the original theory. These features include mailboxes, which store incoming messages, schedulers, which help manage the execution of actors in multi-core systems, actor references, which are special types to help identify actors, and networking components, which enable actors to communicate across multinode systems.

Nevertheless, implementations of the actor model operate largely in the same way. Users create actors and define the types of messages that can be received and the responses to them. Messages consist of any number of parameters of any type supported by the programming language and invoke specific message handlers that match the message signature (i.e., the types of parameters it contains) defined within the receiving actor. The receiving actor can then be programmed to send messages to other actors, create new actors, update its internal state, or change the way it responds to future messages.

### Related work

Parallelization of the SW algorithm can be implemented on different levels: single-instruction, multiple-data (SIMD) parallelism, thread-level parallelism, process-level parallelism, and heterogeneous parallelism.

#### SIMD parallelism

SIMD parallelism, also known as vector-level parallelism, employs a controller to manage multiple processors that simultaneously execute the same process on different data groups (Barry and Crowley 2012). Parasail (Daily 2016), SeqAn (Rahn *et al.* 2018), SWIPE (Rognes 2011), KSW (Li 2018) and its improved version KSW2 (Suzuki and Kasahara 2018) are the most prominent implementations that use this approach. Parasail is a SIMD C (C99) library for implementations of the NW (global), SW (local), and semiglobal sequence alignments. SeqAn uses parallel computing on multiple levels, including data parallelism and vectorization, to provide a versatile library that supports various bioinformatics applications. SWIPE optimizes the SW algorithm to exploit SIMD for faster sequence alignments at the intraalignment parallelization level. KSW and KSW2 are SIMD-based semiglobal alignment algorithms that accelerate the SW algorithm by computing the differences between adjacent cells in the score matrix rather than calculating and storing all the values in the matrix. These SIMD-based approaches demonstrate significant improvements in computational efficiency. For instance, Ahmed *et al.* (2019) reported that SeqAn was 1.58×, 2.5×, and 1.66× faster than Parasail for global, local, and semiglobal alignments, respectively.

#### Thread-level parallelism

Another approach to parallelism applicable to sequence alignment is thread-level approaches. POSIX threads (Pthreads) and OpenMP are examples of technologies that facilitate this type of parallelism. Pthreads offer a portable and flexible model for handling shared-memory parallelism, enabling the creation and management of multiple threads within a single process. On the other hand, OpenMP provides a simpler, more scalable approach to shared-memory model programming by using compiler directives that allow the easy parallelization of code across multiple threads. Different algorithms employ these techniques to enhance the performance of the SW algorithm. SWIPE is specifically designed for systems with shared-memory architectures and uses thread-level parallelization to achieve interalignment parallelism. Daily (2016) reported that SWIPE was between 1.2× and 2.4× faster than Parasail for short sequences. However, Parasail outperformed SWIPE for longer sequences.

#### Process-level parallelism

MPI is a process-level parallelism approach predominantly utilized in distributed-memory systems. This approach operates on the principle that each process maintains an independent stack and code segment during parallel execution. As separate programs, the interaction between processes is facilitated through explicit calls to communication functions, allowing for data exchange and synchronization across different computational nodes. The use of MPI enables sequence alignment to be distributed to multiple nodes, significantly reducing the workload of a single node. Khaled *et al.* (2016) used MPI to develop a hybrid model for sequence alignment. They demonstrated that their model achieved a speed up of 50× over the sequential implementation of SW algorithm using eight nodes with 24 cores each.

#### Heterogeneous parallelism

Computer clusters often feature a mix of high-performance computational units that enable heterogeneous parallelism, where specialized units perform specific tasks, in contrast with the more common homogeneous parallelism, where units are identical. Heterogeneous systems typically include standard processors alongside specialized units like field-programmable gate arrays (FPGAs), graphics processing units (GPUs), and Xeon Phi processors. ADEPT (Awan *et al.* 2020), ManyMap (Feng *et al.* 2019), CUDASW++ (Liu *et al.* 2013), and CUDAlign 3.0 (Sandes *et al.* 2014) leverage GPU technology to significantly enhance the efficiency of sequence alignment processes. Through the use of GPUs, these tools achieve shorter computation times compared with traditional CPU-based implementations. SWIFOLD (Rucci *et al.* 2018) and OSWALD (Rucci *et al.* 2018) employ FPGA technology to enhance the performance of the SW for DNA and protein sequences, respectively. SWAPHI-LS (Liu *et al.* 2014), XSW (Wang *et al.* 2014), and SWIMM (Rucci et al. 2015) utilize Xeon Phi coprocessors to enhance their computational capabilities for sequence alignment. Compared with CUDAlign, CUDASW++, and SWAPHI-LS, XSW achieves much better performance in terms of giga cells per second (GCUPS; Wang *et al.* 2014).

## Materials and methods

### Datasets

To assess the performance of SW-actors against other benchmark implementations, we utilized four datasets retrieved from National Center for Biotechnology Information (NCBI) (Sayers *et al.* 2022): (i) breast cancer gene 1 (BRCA1), consisting of 300 DNA sequences with an average length of 6225 nucleotides; (ii) breast cancer gene 2 (BRCA2), consisting of 659 DNA sequences with an average length of 10 617 nucleotides; (iii) Titin, consisting of 700 DNA sequences with an average length of 61 906 nucleotides; and (iv) a heterogeneous dataset consisting of sequences from multiple genes with an average length of 39 562 nucleotides, generated from a random selection of genes with lengths ranging from 500 to 75 000 nucleotides. All four datasets are available from the SW-actors repository (see Data Availability). The sizes of these datasets are summarized in Table 1.

**Table 1.** Dataset sizes.

Datasets	Number of sequences	**Minimum length (nt)** ^a^	Maximum length (nt)	Average length (nt)
Breast cancer gene 1 (BRCA1)	300	504	10,496	6,225
Breast cancer gene 2 (BRCA2)	659	85	17,977	10,617
Titin	700	52,968	74,778	61,906
Heterogeneous	583	716	74,675	39,562

a nt, nucleotides.

### Parallelization

**Figure 2.**
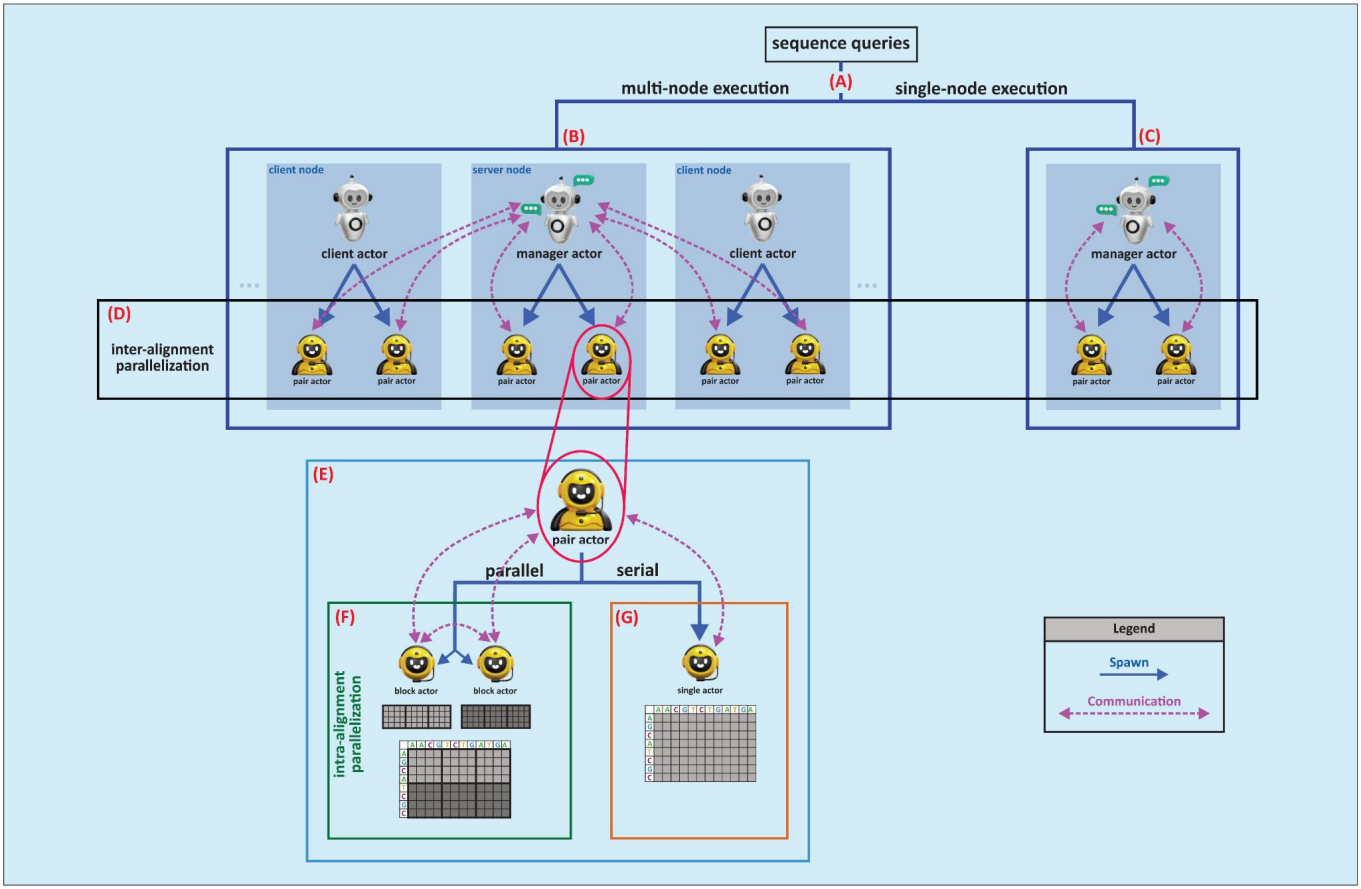
The workflow of SW-actors for sequence alignment. (A) Multinode or single-node execution. (B) Multinode execution: one node operates as the server, spawning a *manager actor*, while the remaining nodes operate as clients, each spawning *client actor*s. Both *manager* and *client actor*s spawn *pair actors*. Only the *manager actor* communicates with the *pair actor*s, sending alignment tasks and receiving their results. (C) Single-node execution: The *manager actor* spawns *pair actor*s and manages communication by sending alignment tasks and receiving alignment results. (D) Interalignment parallelization: concurrent spawning of multiple *pair actor*s. (E) Score matrix: parallel or serial construction. (F) Intraalignment parallelization: *block actor*s are spawned to handle row blocks of the score matrix in parallel using an anti-diagonal strategy starting from the top-left; once a *block actor* completes its assigned row block, it reports the maximum score to its *pair actor* and processes another row block. (G) Serial computation: A *single actor* processes the score matrix row-by-row as a single matrix.

Our primary objective was to fully leverage the benefits of parallelization, thereby enhancing the overall efficiency of the sequence alignment. To achieve this objective, we conducted a comprehensive analysis of different parallelization algorithms on the interalignment and intraalignment levels to determine the optimal parallelization configuration; the details of the results are described in Supplementary Section S1. Figure 2 illustrates the workflow of SW-actors in detail for sequence alignment with multinode and single-node executions at both the interalignment and intraalignment parallelization levels.

#### Interalignment parallelization

To begin, we evaluated the effectiveness of OpenMP, MPI, and actors in parallelizing the alignment of sequence pairs (Supplementary Section S1.1). This approach allows us to exploit multiple processing units, significantly reducing the time required for multiple sequence comparisons.

#### Intraalignment parallelization

To further increase the performance of the SW algorithm, we implement the computation of the score matrix using OpenMP, MPI, and actors (Supplementary Section S1.2). This parallelization focuses on accelerating the individual sequence alignment processes by parallelizing the computation-intensive tasks of computing the score matrix and traceback steps.

### Experiments and evaluation metrics

We ran four competing implementations, SW-actors, Parasail (v2.6.2), SeqAn (v3.3.0), and SWIPE (v2.1.1), on the four datasets described in Table 1 with the scoring scheme: match=2, mismatch=−1, and gap=−2.

All implementations are written in C++. For the implementation involving actors, we used CAF (Charousset *et al.* 2014). For implementations involving OpenMP and MPI, we used the omp.h (Dimakopoulos *et al.* 2003) and mpi.h (Snir 1998) libraries, respectively. The source codes for the third-party implementations were obtained from their respective repositories, i.e., https://github.com/jeffdaily/parasail, https://github.com/seqan, and https://github.com/torognes/swipe. The compiler used for the implementation was GNU g++ version 9.3.0, and the compiler flag was set to -std=c++17 -O3 for optimization.

We used a compute node equipped with two Intel Xeon Gold 6148 CPUs, with 40 cores running at 2.40 GHz with 185 GB of RAM under a Linux operating system with varying numbers of processors (see Supplementary Section S2). To evaluate the performance of the competing implementations, the wall-clock time, speedup, and memory usage are reported.

## Results

In this section, the results of the experiments carried out are described, and the performance of SW-actors is compared with the existing benchmark implementations Parasail, SeqAn, and SWIPE. A scaling analysis using speedup has also been conducted to assess how each implementation performs with varying numbers of processors. The outputs of the competing algorithms are generated using the same format, where applicable.

### Performance evaluation

#### Wall-clock time

**Figure 3.**
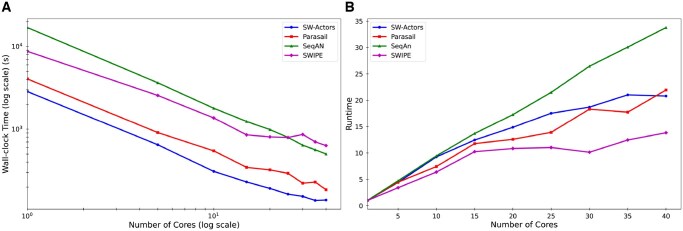
(A) Wall-clock time and (B) speedup of competing implementations across varying numbers of cores for the breast cancer gene 1 (BRCA1) dataset.

**Figure 4.**
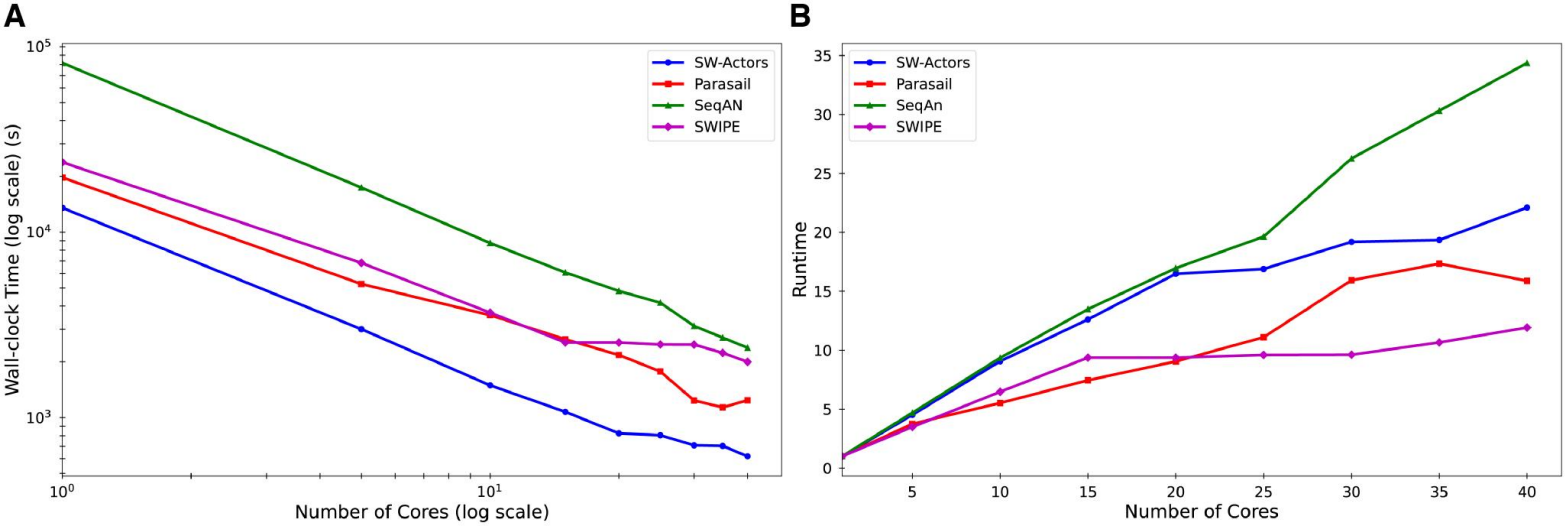
(A) Wall-clock time and (B) speedup of competing implementations across varying numbers of cores for the breast cancer gene 2 (BRCA2) dataset.

We first evaluated the performance of the competing implementations in terms of wall-clock time. As detailed in Table 2 and illustrated in Figs. 3A and 4A, the results demonstrate that SW-actors consistently outperforms the other implementations in terms of wall-clock time across the four datasets. For the BRCA1 dataset, SW-actors was 1.33× faster than the next best-performing benchmark implementation in terms of wall-clock time. For the BRCA2 dataset, SW-actors was 2.00× faster than the next best-performing benchmark implementation. As summarized in Table 2, SW-actors outperformed all the other implementations. In particular, SW-actors achieved 2.49× and 1.94× faster wall-clock times for the largest (Titin and heterogeneous) datasets. Despite the computational challenges posed by the dataset size, SW-actors maintained computational efficiency, showing its ability in handling longer sequences. This performance underscores the effectiveness of the actor model in sequence alignment as a solution for accelerating the SW algorithm. The values in Table 2 represent the total wall-clock time, including both computation and I/O. A breakdown of I/O and computation times is provided in the Supplementary Section S2.

**Table 2.** Performance of competing implementations using 40 cores for different datasets.

Dataset	Implementation	**Wall-clock time (s)** ^a^	**Speedup (** × **)** ^b^	**Memory usage (GB)** ^c^
Breast cancer gene 1 (BRCA1)	SW-actors	**139.40** ^d^	20.81	16.46
	Parasail	185.80	21.95	97.07
	SeqAn	501.40	**33.78**	4.06
	SWIPE	633.00	13.83	**0.05**
Breast cancer gene 2 (BRCA2)	SW-actors	**619.00**	22.10	49.54
	Parasail	1241.00	15.88	127.63
	SeqAn	2382.40	**30.31**	10.41
	SWIPE	1999.40	9.62	**0.07**
Titin	SW-actors	**52 977**	NA^e^	833.58
	Parasail	132 167	NA	1679.36
	SeqAn	228 536	NA	486.94
	SWIPE	192 283	NA	**0.26**
Heterogeneous	SW-actors	**16 567**	NA	831.23
	Parasail	31 971	NA	1 64 080
	SeqAn	62 696	NA	466.50
	SWIPE	62 683	NA	**0.23**

a s, seconds.

b × , speedup factor (times).

c GB: gigabytes.

d The best values for the performance metrics are in boldface.

e Given the significantly longer execution times required for Titin and heterogeneous datasets, scalability analysis was infeasible with available resources.

#### Speedup

We evaluated the scalability performance of the competing implementations using the speedup (see Supplementary Section S2). As detailed in Table 2 and illustrated in Figs. 3B and 4B, the results confirm that SW-actors shows good strong scaling; i.e., increasing the number of processors increases the speedup in rough proportion. The results show SW-actors is the second-best performing implementation in terms of scalability and scales efficiently with the increasing number of processors. The results demonstrate that SW-actors achieved a speedup of 20.81× on 40 cores compared with using a single processor for the BRCA1 dataset and a speedup of 22.10× for the BRCA2 dataset. Nominally, SeqAn achieved the best speedup for both BRCA1 and BRCA2. However, its single-core implementation had a much longer wall-clock time, and this resulted in a large relative speedup but which was accompanied by large wall-clock times. SW-actors, which performs efficiently even in a single-core setting, did not exhibit as significant a speedup in the parallel execution phase. These results suggest that SW-actors exhibits balanced performance across both single-core and multi-core architectures.

#### Memory usage

Memory usage is an important metric that reflects the amount of system memory (RAM) utilized by a piece of software during execution. Efficient memory management is essential for handling large datasets and performing computations. In practice, there is often a trade-off between using more memory and achieving shorter wall-clock times. For sequence alignment scenarios in practice, particularly with larger datasets, the importance of wall-clock time often takes precedence over memory consumption. This is because the speed at which the results are produced is critical for practical applications. The results presented in Table 2 demonstrate that, except for Parasail, SW-actors uses more memory than the competing implementations. This higher memory usage, however, pays off in terms of an ability to deliver faster wall-clock times.

## Discussion

This article introduces an accelerated approach to local sequence alignment that leverages the actor model of concurrent computation. The approach parallelizes the SW algorithm at both the interalignment and intraalignment levels. To the best of our knowledge, this represents the first implementation of the actor model to enhance sequence alignment efficiency. Our findings confirm that SW-actors consistently demonstrates superior performance in terms of wall-clock time across four diverse datasets, outperforming the competing implementations for all dataset sizes and complexities considered. This performance underscores the robustness and efficiency of SW-actors. Its success lies in the efficiency of actor models in task scheduling as a key factor in the observed shorter wall-clock times. Actors manage concurrent tasks through asynchronous message passing, allowing for effective distribution of alignment tasks (interalignment level) and score matrix computation (intraalignment level) across multiple processors. This approach minimizes coordination overhead and maximizes parallelism, enabling each processor to handle alignment tasks independently. Consequently, SW-actors benefits from reduced idle times and improved resource utilization, resulting in significantly shorter wall-clock times.

Although SW-actors exhibits the second-highest memory usage among the methods tested and shows a slightly smaller speedup compared with the leading implementation, it clearly stands out as the fastest overall implementation in terms of wall-clock time.

SW-actors has several limitations, and there are some directions for future research. One primary limitation is the memory usage, which could pose challenges when scaling to extremely large datasets or operating within memory-constrained environments. Additionally, scalability could perhaps be further improved. Future work could explore hybrid approaches that integrate the use of the actor model with more memory-efficient algorithms. Furthermore, applying the actor model to other alignment algorithms, such as the NW algorithm, could broaden the applicability and effectiveness of the approach.

## Conclusion

The SW algorithm remains a leading implementation for sequence alignment due to its accuracy in performing local alignments. However, its computational intensity necessitates parallel implementations to achieve practical wall-clock times. As larger and larger datasets with biological sequences are continually being released, efficient tools for handling larger datasets have become increasingly important. The novel approach of implementing the SW algorithm using the actor model for parallel execution of local sequence alignment shows significant improvements in performance compared with the traditional OpenMP and MPI paradigms. SW-actors stands out for its superior wall-clock time performance, setting the stage for future advancements in sequence alignment, where balancing efficiency, resource utilization, and fault tolerance in an automated fashion are prerequisites for practical success. These performance improvements can be attributed to the use of the actor model, which avoids job synchronization and reduces the amount of memory allocation and deallocation overhead associated with traditional parallel programming paradigms such as OpenMP or MPI. More specialized optimizations (e.g., SIMD) are left for future work and are expected to yield further performance gains.

## Supplementary Material

vbaf173_Supplementary_Data

## Data Availability

The SW-actors source code and underlying data underlying are available at https://git.cs.usask.ca/numerical_simulations_lab/actors/papers/SW-actors.
