# Supplementary material for: SW-actors: accelerating the Smith–Waterman algorithm via actors
Source: Bioinform Adv. 2025 Jul 28;5(1):vbaf173. doi: 10.1093/bioadv/vbaf173 (PMC12449131; doi:10.1093/bioadv/vbaf173)
Supplement: vbaf173_Supplementary_Data [file vbaf173_supplementary_data.pdf]

SW-Actors: Accelerating the Smith–Waterman Algorithm via Actors
Supplementary data

Reza Rafati Bonab, Ali Akbar Jamali, Kyle Klenk, Mohammad Mahdi Moayeri, Raymond J. Spiteri

S1 Bi-level Parallelization

S1.1 Inter-alignment parallelization

We employed various parallel computing techniques, including OpenMP, MPI, and the actor model, to evaluate their effectiveness in parallelizing the alignment of sequence pairs using Smith-Waterman (SW) (Smith and Waterman, 1981) algorithm. As shown in Table S1, the results demonstrate that the actor model achieves the best performance in terms of wall-clock time for both BRCA1 and BRCA2 datasets.

Table S1: Performance (wall-clock time (s)) of different algorithms for Inter-alignment parallelization on 40 cores using BRCA1 and BRCA2 datasets.

Algorithm	BRCA1	BRCA2
Actor	**139.40**[1]	**619.00**
MPI	282.00	1,289.60
OpenMP	192.60	2,820.00

[1] The best values for the performance metric are in **boldface**.

S1.2 Intra-alignment parallelization

To enhance the performance of the SW algorithm, we implemented the construction of the alignment matrix using parallel computing techniques, including OpenMP, MPI, and the actor model. This approach aims to accelerate individual sequence alignment processes by parallelizing score matrix construction. As summarized in the Table S2, the results showed that for matrix-filling parallelization, the OpenMP model outperformed both actors and MPI in terms of wall-clock time.

Table S2: Performance (wall-clock time (s)) of different algorithms for intra-alignment parallelization using two sequences with 51K and 53K nucleotides.

Algorithm	Wall-clock time
Actor	7.10
MPI	15.60
OpenMP	**5.20**[1]
Serial(row-by-row)	13.70
Serial(anti-diagonal)	48.50

[1] The best value for the performance metric is in **boldface**.

S1.3 Bi-level parallelization

To investigate the performance of the hybrid parallelized method based on the best methods in inter- and intra-alignment parallelization, we implemented the actor model for inter-alignment parallelization and OpenMP for intra-alignment parallelization. As depicted in Table S3, the results showed that this

hybrid parallelized method did not achieve the best performance. According to these results we selected the hybrid model including actor model for inter-alignment parallelization and serial (row-by-row) for intra-alignment parallelization as the best method for further analyses.

Table S3: Performance (wall-clock time(s)) of different hybrid methods on 40 cores using BRCA1 and BRCA2 datasets.

**Inter-alignment parallelization**	**Intra-alignment parallelization**	**BRCA1**	**BRCA2**
Actor	Serial (row-by-row)	**139.40**[1]	**619.00**
	OpenMP	990.40	7,506.80
	Actor	261.00	1,232.00
MPI	Serial (row-by-row)	282.00	1,289.60
	OpenMP	702.60	3,093.00
OpenMP	Serial (row-by-row)	192.80	2,820.00
	OpenMP	451.60	6,991.80

[1] The best values for the performance metrics are in **boldface**.

S2 Multi-core Performance Analysis

In this study, we conducted further analysis to evaluate the performance of different algorithms including Parasail (Daily, 2016), SeqAn (Rahn et al., 2018), and SWIPE (Rognes, 2011) using different numbers of cores. We ran each algorithm with a range of cores (1, 5, 10, 15, ..., 40) to investigate their scalability and efficiency. To ensure the reliability of our results, we ran each configuration five times and reported the average performance in Table S4 and Table S5 for the BRCA1 and BRCA2 datasets. Due to the significantly longer execution time associated with the Titin dataset, it was not feasible to conduct the same analysis for this dataset within the available resources.

Table S4: Performance of competing algorithms on different number of cores using BRCA1 dataset.

**Metric**	**Algorithm**	**I/O Time**	**Number of cores**								
			**1**	**5**	**10**	**15**	**20**	**25**	**30**	**35**	**40**
Wall-clock time (s)[1]	SW-Actors	Yes	**2,856.00**[2]	**648.20**	**309.40**	**230.40**	**192.60**	**164.20**	**154.20**	**137.80**	**139.40**
		No	**2,766.40**	**614.80**	**306.40**	**232.60**	**193.60**	**168.00**	**154.40**	**141.40**	**136.60**
	Parasail	Yes	4,058.00	910.20	547.00	345.80	322.60	293.00	222.40	229.60	185.80
		No	3,684.60	1,061.20	547.40	388.40	274.00	286.00	213.80	195.20	180.20
	SeqAN	Yes	16,923.20	3,646.00	1,797.00	1,240.80	990.00	794.80	640.40	563.40	501.40
		No	15,687.00	3,265.40	1,686.00	1,125.80	900.20	718.80	603.40	511.00	453.00
	SWIPE	Yes	8,738.20	2,565.00	1,369.60	853.80	806.40	793.20	863.20	702.80	633.00
		No	8,620.00	2,446.00	1,279.60	844.20	827.60	814.20	810.60	733.20	630.20
SpeedUp (X)[3]	SW-Actors	Yes	1.00	4.41	9.25	12.45	14.87	17.54	18.71	21.03	20.81
		No	1.00	4.69	9.04	11.92	14.38	16.60	18.12	19.84	20.58
	Parasail	Yes	1.00	4.46	7.42	11.77	12.58	13.90	18.32	17.75	21.95
		No	1.00	3.47	6.74	9.51	13.45	12.92	17.30	18.88	20.46
	SeqAN	Yes	1.00	**4.72**	**9.44**	**13.69**	**17.28**	**21.50**	**26.46**	**30.08**	**33.78**
		No	1.00	**4.80**	**9.30**	**13.93**	**17.42**	**21.83**	**26.00**	**30.70**	**34.63**
	SWIPE	Yes	1.00	3.41	6.38	10.24	10.84	11.03	10.13	12.45	13.83
		No	1.00	3.52	6.74	10.21	10.42	10.59	10.64	11.76	13.69
Memory usage (GB)[4]	SW-Actors	Yes	0.51	2.06	4.12	6.17	8.23	10.28	12.34	14.40	16.46
		No	0.42	2.06	4.12	6.17	8.23	10.29	12.34	14.40	16.45
	Parasail	Yes	93.12	97.39	97.29	93.14	99.16	98.46	99.07	98.90	97.07
		No	93.13	96.26	97.06	98.11	97.60	98.21	95.87	100.46	98.84
	SeqAN	Yes	3.40	4.09	4.00	3.95	3.35	3.33	3.08	3.75	4.06
		No	2.94	3.21	3.22	3.12	3.17	3.15	3.20	3.16	3.19
	SWIPE	Yes	**0.01**	**0.03**	**0.02**	**0.02**	**0.03**	**0.03**	**0.04**	**0.04**	**0.05**
		No	**0.01**	**0.03**	**0.02**	**0.02**	**0.03**	**0.03**	**0.04**	**0.04**	**0.05**

[1] **s:** seconds.
[2] The best values for the performance metrics are in **boldface**.
[3] **X:** Speedup factor (times).
[4] **GB:** gigabytes.

Table S5: Performance of competing algorithms on different number of cores using BRCA2 dataset.

**Metric**	**Algorithm**	**I/O Time**	**Number of cores**								
			**1**	**5**	**10**	**15**	**20**	**25**	**30**	**35**	**40**
Wall-clock time (s)[1]	SW-Actors	Yes	**13,533.20**[2]	**3,001.20**	**1,494.80**	**1,074.80**	**823.60**	**804.60**	**709.40**	**704.40**	**619.00**
		No	**12,837.80**	**3,095.40**	**1,393.20**	**1,027.60**	**859.20**	**743.60**	**755.80**	**674.00**	**561.00**
	Parasail	Yes	19,710.00	5,259.40	3,570.80	2,647.00	2,178.00	1,775.80	1,237.40	1,136.80	1,241.00
		No	20,261.20	4,625.20	3,121.20	2,564.40	2,286.80	1,526.60	1,330.80	1,139.60	1,040.40
	SeqAN	Yes	81,718.60	17,413.00	8,747.00	6,062.80	4,822.40	4,167.80	3,121.40	2,700.00	2,382.40
		No	72,155.60	15,971.00	7,691.40	5,670.20	4,232.20	3,326.40	2,769.20	2,435.80	2,123.20
	SWIPE	Yes	23,824.20	6,831.60	3,685.00	2,543.40	2,542.80	2,482.00	2,477.20	2,235.20	1,999.40
		No	23,518.20	6,909.20	3,713.60	2,534.40	2,508.00	2,443.00	2,438.00	2,196.00	1,969.00
SpeedUp (X)[3]	SW-Actors	Yes	1.00	4.51	9.06	12.61	16.48	**16.89**	19.19	19.35	22.10
		No	1.00	4.15	9.22	12.51	14.98	17.34	17.10	19.20	23.14
	Parasail	Yes	1.00	3.75	5.52	7.45	9.05	11.10	15.93	17.34	15.88
		No	1.00	4.38	6.49	7.90	8.86	13.28	15.24	17.78	19.48
	SeqAN	Yes	**1.00**	**4.69**	**9.35**	**13.48**	**16.96**	19.63	**26.26**	**30.31**	**34.36**
		No	**1.00**	**4.52**	**9.38**	**12.73**	**17.05**	**21.69**	**26.06**	**29.62**	**33.98**
	SWIPE	Yes	1.00	3.49	6.47	9.37	9.37	9.60	9.62	10.66	11.92
		No	1.00	3.40	6.33	9.28	9.38	9.63	9.65	10.71	11.94
Memory usage (GB)[4]	SW-Actors	Yes	1.78	7.44	13.41	19.45	25.07	31.50	37.50	43.47	49.54
		No	1.22	6.04	12.06	18.09	24.12	30.14	36.17	42.20	48.23
	Parasail	Yes	111.75	129.80	129.94	128.57	126.24	128.16	127.05	128.84	127.63
		No	111.76	127.57	127.82	127.64	127.18	127.56	128.60	126.46	126.53
	SeqAN	Yes	10.41	10.24	10.39	10.39	10.39	10.24	10.35	10.40	10.41
		No	10.12	10.35	10.38	10.34	10.37	10.34	10.39	10.36	10.37
	SWIPE	Yes	**0.01**	**0.02**	**0.03**	**0.04**	**0.04**	**0.05**	**0.06**	**0.07**	**0.07**
		No	**0.01**	**0.02**	**0.03**	**0.04**	**0.04**	**0.05**	**0.06**	**0.07**	**0.07**

[1] **s:** seconds.

[2] The best values for the performance metrics are in **boldface**.

[3] **X:** Speedup factor (times).

[4] **GB:** gigabytes.

S3 Multi-node Performance Analysis

In our study, we evaluated the performance of SW-Actors, against the SWIPE algorithm within a multi-node, multi-core configuration. As depicted in Table S6 and Table S7, the results demonstrate that SW-Actors outperforms SWIPE across various metrics, including wall-clock time, and speedup. Specifically, SW-Actors consistently achieved lower execution times as the number of cores increased. In contrast, SWIPE exhibited significant overhead, particularly in multi-node environments, which adversely affected its scalability and overall performance. This overhead resulted in reduced efficiency, underscoring the limitations of SWIPE in distributed computing scenarios. The enhanced performance of SW-Actors can be attributed to its resource management and reduced communication latency. These findings highlight the potential of SW-Actors to significantly improve computational efficiency and scalability in complex, multi-core, and multi-node systems.

It is important to note that Parasail and SeqAn, which are based on OpenMP, cannot be run on multiple nodes. OpenMP is limited to single-node environments because it relies on shared memory for communication between threads. Distributed-memory systems (multiple nodes) require communication mechanisms to pass data between nodes, which OpenMP cannot handle.

Table S6: Performance of different algorithms on different number of cores on distributed nodes using BRCA1 dataset.

**Algorithm**	**Nodes**	**CPU per node**	**Total CPUs**	**Wall-clock time (s)**[1]	**Speedup (×)**[2]	**Memory usage (GB)**[3]
SW-Actors	1	1	1	2,856.00	1.00	0.51
	1	40	40	139.40	20.81	16.46
	2	20	40	103.40	28.16	8.23
	2	40	80	76.80	38.80	16.46
	4	10	40	102.40	32.23	4.12
	4	20	80	56.40	52.69	8.23
	4	40	160	45.40	67.03	16.46
SWIPE	1	1	1	8,738.20	1.00	0.01
	1	40	40	633.00	13.83	0.05
	2	20	40	702.60	12.44	0.05
	2	40	80	428.00	20.43	0.09
	4	10	40	1,123.80	7.78	0.05
	4	20	80	740.40	11.81	0.09
	4	40	160	520.40	16.80	0.14

[1] **s:** seconds.
[2] **X:** Speedup factor (times).
[3] **GB:** gigabytes.

Table S7: Performance of different algorithms on different number of cores on distributed nodes using BRCA2 dataset.

**Algorithm**	**Nodes**	**CPU per node**	**Total CPUs**	**Wall-clock time (s)**[1]	**Speedup** ($\times$)[2]	**Memory usage (GB)**[3]
SW-Actors	1	1	1	13,533.20	1.00	1.78
	1	40	40	619.00	22.13	49.54
	2	20	40	464.60	29.29	24.12
	2	40	80	363.20	37.93	48.23
	4	10	40	413.40	32.85	12.07
	4	20	80	246.80	55.41	24.13
	4	40	160	185.60	75.51	48.25
SWIPE	1	1	1	23,822.04	1.00	0.01
	1	40	40	1,999.07	11.92	0.07
	2	20	40	2,131.50	11.18	0.07
	2	40	80	1,299.40	18.34	0.17
	4	10	40	3,061.70	7.78	0.07
	4	20	80	1,967.60	12.12	0.12
	4	40	160	1,362.80	17.49	0.23

[1] **s:** seconds.
[2] **X:** Speedup factor (times).
[3] **GB:** gigabytes.

References

Daily, J. (2016, Feb). Parasail: SIMD C library for global, semi-global, and local pairwise sequence alignments. *BMC Bioinformatics 17*(1), 81.

Rahn, R., S. Budach, P. Costanza, M. Ehrhardt, J. Hancox, and K. Reinert (2018, 05). Generic accelerated sequence alignment in SeqAn using vectorization and multi-threading. *Bioinformatics 34*(20), 3437–3445.

Rognes, T. (2011, Jun). Faster Smith–Waterman database searches with inter-sequence SIMD parallelisation. *BMC Bioinformatics 12*(1), 221.

Smith, T. F. and M. S. Waterman (1981). Identification of common molecular subsequences. *Journal of Molecular Biology 147*(1), 195–197.
